# The Mechanical Side of Anatomical Articulation

**Published:** 1912-04-15

**Authors:** 


THE MECHANICAL SIDE OF ANATOMICAL ARTICULATION. This is a collection of the contributions of Dr. George W. Clapp, to the Dental Digest, on the subject of articulating artificial teeth. It gives a very comprehensive statement of the principles involved and the technical procedures of bite taking and adjustment of the teeth in their anatomical relations so as to secure pacticable occlusion. It makes a valuable contribution to our literature on this subject and we are glad to have it reprinted in book form. It would be a good idea for every dentist to obtain a copy of this little book that he may at least get interested in improving his present inefficient prosthetic methods.

